# 38081 A Whole Blood Signature of Neutrophil Expression and Adaptive Immune Downregulation Characterizes Sepsis Mortality

**DOI:** 10.1017/cts.2021.662

**Published:** 2021-03-30

**Authors:** H.M. Giannini, C.V. Cosgriff, X.M. Lu, J.R. Reilly, B.J. Anderson, T.K. Jones, C.A.G. Ittner, A.R. Weissman, T. Agyeum, T. Dunn, M. Shashaty, N.J. Meyer

**Affiliations:** 1 Institute for Bioinformatics, University of Pennsylvania; 2 All authors are from the Division of Pulmonary and Critical Care Medicine, Perelman School of Medicine, University of Pennsylvania, Philadelphia, PA unless otherwise noted

## Abstract

ABSTRACT IMPACT: This work identifies host immune perturbations in sepsis mortality that suggests targets for a precision medicine paradigm in the host response to infection. OBJECTIVES/GOALS: To compare the early whole blood transcriptome during sepsis between 30-day survivors and non-survivors in the Intensive Care Unit (ICU), and to evaluate for pathway enrichment that might explain sepsis lethality. METHODS/STUDY POPULATION: We enrolled 162 sepsis patients in the first 24 hours of ICU admission, particularly targeting individuals requiring vasopressors. Peripheral whole blood was collected in PAXgene vacutainers. Isolated RNA was analyzed with Affymetrix Human Genome ST 2.0 microarray. Differential gene expression was performed with Bioconductor/R/limma using log2 fold-change +/-0.6 as a threshold for differential expression, and a Benjamini-Hochberg adjusted p-value <0.05 to declare significance. Functional gene enrichment was performed using the Gene Ontology (GO) database with PANTHER overrepresentation test (Fisher’s Exact) on all transcripts with adjusted p-value <0.05. Pathways analysis was performed with the Reactome Project using the raw fold change and significance data to identify dysregulated pathways. RESULTS/ANTICIPATED RESULTS: There were 58 non-survivors (36% mortality). We identified 39 genes as differentially expressed between sepsis non-survivors and survivors; 31 were upregulated in non-survivors and 8 had reduced expression. Several of the most overexpressed transcripts are neutrophil-specific, including LCN, MPO, OLF4M4, DEFA3, and DEFA4. A functional gene overrepresentation test further supports this finding, as the most enriched gene ontologies were neutrophil-mediated killing, neutrophil cytotoxicity, neutrophil extravasation, and respiratory burst, all demonstrating higher than 10-fold enrichment and FDR < 0.02. Pathway analysis of the peripheral blood transcriptome was notable for immune response derangement, specifically downregulation of both innate and adaptive immune pathways (FDR < 0.00001). DISCUSSION/SIGNIFICANCE OF FINDINGS: We identified increased expression of neutrophil-related genes in sepsis non-survivors, replicating candidates previously identified in pediatric sepsis mortality and ARDS. These immune perturbations in sepsis mortality may represent key targets for eventually employing precision medicine strategies in sepsis.

